# A Novel Porcine Model for the Study of Cerebrospinal Fluid Dynamics: Development and Preliminary Results

**DOI:** 10.3389/fneur.2019.01137

**Published:** 2019-10-23

**Authors:** David Fleischman, Omkar Kaskar, Rayad Shams, Xinxin Zhang, Daniel Olson, Carlton Zdanski, Brian D. Thorp, Andrey V. Kuznetsov, Landon Grace, Yueh Z. Lee

**Affiliations:** ^1^Department of Ophthalmology, University of North Carolina at Chapel Hill, Chapel Hill, NC, United States; ^2^Department of Mechanical and Aerospace Engineering, NC State University, Raleigh, NC, United States; ^3^Department of Radiology, University of North Carolina at Chapel Hill, Chapel Hill, NC, United States; ^4^Department of Otolaryngology, Head & Neck Surgery, University of North Carolina at Chapel Hill, Chapel Hill, NC, United States

**Keywords:** CSF, CSF pressure, optic canal, glaucoma, papilledema, space-flight associated neuro-ocular syndrome

## Abstract

Idiopathic intracranial hypertension, space-flight associated neuro-ocular syndrome (SANS), and glaucoma are conditions that are among a spectrum of cerebrospinal fluid (CSF)-related ophthalmologic disease. This implies that local CSF pressures at the level of the optic nerve are involved to variable extent in these disease processes. However, CSF pressure measurements are problematic due to invasiveness and interpretation. The pressure measured by a lumbar puncture is likely not the same as the orbital CSF pressure. It is believed this is at least in part due to the flow restrictive properties of the optic canal. To investigate CSF flow within the orbit, a model for CSF dynamics was created using three medium-sized pigs. Contrast was administered through a lumbar subarachnoid space access. The contrast front was imaged with repeated computed tomographic (CT) imaging. Once contrast entered the orbit, rapid, sequential CT imaging was performed until the contrast reached the posterior globe. Head tilting was performed to highlight the role of gravitational dependence within the subarachnoid space.

## Introduction

The primary circulating fluid of the central nervous system is the cerebrospinal fluid (CSF). Produced in the choroid plexus within the lateral, third and fourth ventricles, the fluid takes a circuitous route through the spinal column and throughout the cerebral cortex. The optic nerves themselves are surrounded by CSF enclosed by the dura mater, or the optic nerve sheath. Therefore, the CSF and its flow is important in the context of ophthalmologic disease. For example, idiopathic intracranial hypertension (IIH), an uncommon disease that primarily affects young, obese women, is characterized by increased intracranial pressure in the absence of a secondary or identifiable cause. With the development of papilledema, swelling of the optic nerve, the condition can lead to significant vision loss and blindness. Cerebrospinal fluid pressure (CSFP) has re-emerged as a possible risk factor for glaucoma. Space flight-associated neuro-ocular syndrome (SANS), a condition that has affected astronauts returning from prolonged microgravitational exposure, can produce optic nerve swelling, hyperopia, and globe flattening (1).

One major problem in the investigation of CSF-related ophthalmologic diseases is the difficulty in studying this variable. The gold standard for CSFP measurement is a lumbar puncture, a procedure that is uncomfortable and is not without risk. Further, the pressure measurement is performed in the lateral decubitus or prone position, in which humans do not spend much of their lifetime, unless bed-ridden, in a position similar to this. This is important since the relationship between the lumbar CSFP and intracranial pressure is not always equivalent. In the decubitus position, it has been shown that the CSFP in the lateral ventricle is similar to the pressure measured by lumbar puncture (2). However, when upright, the intracranial pressure is lower than lumbar spine pressure, and may actually be subatmospheric (3).

Further complicating our understanding of CSFP in ophthalmic disease is the knowledge that the fluid conductivity between the intracranial compartment and the orbital CSF space, or the perioptic subarachnoid space (POSAS), may not be congruous. Studies by Morgan et al. (4–6) and Hou et al. (7) were performed in dogs. Morgan's group (4) identified a direct relationship between orbital and intracranial pressure provided that the pressure was >-0.5 mm Hg. Hou and colleagues (7) found an algorithmic relationship between the POSAS and the intracranial pressure, and when intracranial pressure was reduced by 30%, there was a sudden disconnection between the two compartments, which they termed as the break point. Beyond this point, intracranial pressure no longer had any influence on the orbital CSFP. Liu and Kahn (8) performed this compartmental study in human cadavers. A linear relationship between intracranial and orbital CSF pressure was identified, but within the optic nerve itself a pressure gradient exists in which the pressure closer to the globe is lower than that near the optic canal.

Feola et al. (9), in 3 eyes of their porcine model, found that retrobulbar CSF pressure was important in the biomechanical properties of the lamina cribrosa. This is despite earlier studies by Killer et al. (10, 11) that have suggested that CSFP may not be as important to the pathogenesis of glaucoma as a lack of fluid flow within this space. The optic canal, the bony foramen of the lesser wing of the sphenoid, through which the optic nerve exits the orbital space into the anterior cranial fossa, is intimately related to the optic nerve in this area, affording only a thin area for CSF egress between these two spaces. This functionally acts as a natural flow restrictor and may compartmentalize CSF in the orbital space. Killer found reduced contrast into the POSAS of patients with normal tension glaucoma (NTG) compared to controls after CT myelograms were performed. Despite this, Golzan et al. (12) suggested CSF flow in the perioptic subarachnoid space in humans could reach velocities of up to 50 mm per second as detected by a phase contrast magnetic resonance imaging. Velocities at this scale could explain a theory by Wostyn et al. (13) that the fast circulation of CSF within the perioptic subarachnoid space could be protective against glaucoma by continually evacuating toxic metabolic byproducts.

Boye et al. (14) used an interesting technique to measure relative CSF flow in the optic canal using the MRI phase shift from phase contrast images and calculating a flow-range ratio between the intracranial cavity and the perioptic subarachnoid space. After testing 11 age-matched controls to 15 NTG patients, they found lower flow-range ratios in the NTG group, thereby suggesting impaired CSF dynamics in this disease. While a successful and useful technique and measure, true velocities are not an output of this technique and not a practical measure for invasive CSF studies.

Therefore, the creation of a suitable animal model to investigate CSF dynamics is important. Pigs are docile and easy to handle and have previously been recognized as suitable for research of orbit surgery due to similarities to humans (15). The purpose of this investigation is to describe a novel porcine model for examining CSF flow between the intracranial and orbital compartments in a medium-sized pig model and to suggest its utility in assessing the plausibility of prior studies' CSF velocities and reassessing the interpretation of contrast-infused CSF studies.

## Materials and Methods

**Figure 1:**
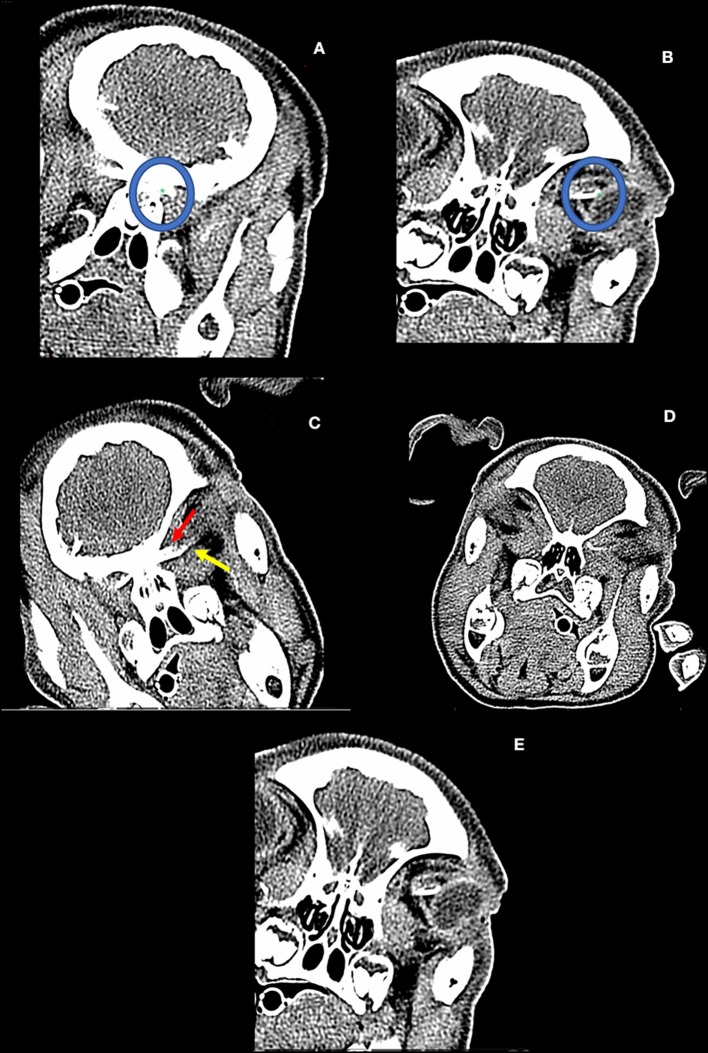
**(A,B)** The dependent optic nerve filling pattern is determined by ROI placement (green dot within blue circles). The three-dimensional linear distance between these two points is determined by the software. Tortuosity of the optic nerve is not incorporated by this technique. **(C)** Pig from experiment #2. First detected entry of contrast into the dependent optic nerve (non-dependent contrast entry not pictured). **(D)** Pig from experiment #3. Note the differential filling of the inferior (yellow line) and superior aspect (red line) of the non-dependent optic nerve. **(E)** Pig from experiment #3: Complete filling of the inferior aspect of the dependent optic nerve with accumulation at the bulbous cul de sac of the nerve adjacent to the left globe.

Three medium sized pigs, *suc scrofa* (30–50 lbs) were used as the model for this work. The study was approved by the institutional IACUC and performed in conjunction with and under direct observation of the Division of Laboratory Animal Medicine personnel. The studies were carried out in strict accordance with the National Institutes of Health guide for the care and use of Laboratory animals (NIH Publications No. 8023, revised 1978). Anesthesia and sedation were administered to minimize discomfort and distress. A lumbar puncture was performed in the animal in the prone position with its legs flexed to separate the intervertebral spaces. A line was drawn between the most cranial aspect of the bilateral wings of the ileum. A 22-gauge 1.5-inch needle was used to access the subarachnoid space. Variable doses of iopamidol (molecular weight 778 D, Bracco, Milano, Italy), were delivered into the subarachnoid space after confirming entry with computed tomographic (CT) imaging. Doses were different between the pigs in an effort to find a concentration that permitted rapid migration of the contrast front to the orbital CSF space. Repeat CT imaging using an orbital imaging protocol was performed to visualize the movement of the contrast agent. If contrast was not migrating, the animal was placed in Trendelenburg position and re-imaged. Contrast and positioning were adjusted on a case-by-case basis as trial-and-error. Once contrast was observed to migrate rostrally, serial CT scans were performed until contrast was visualized abutting the globes. Images were reconstructed using both bone and soft tissue windows to 0.6 mm slices. After the first pig, all other pigs had their head tilted in order to visualize differential contrast migration as a further function of dependence. The position of the contrast front was determined by image analysis through OsiriX MD v 10.0 (Pixmeo SARL, Geneva, Switzerland). Images were primarily analyzed in a coronal view, defined by a plane that was tangential to the optic nerve. A region-of-interest (ROI) was placed at the optic canal and the end of the obvious contrast front. When a superior and an inferior aspect of the nerve sheath were highlighted by the contrast front, a separate ROI was placed at the initiation of the nerve from the optic canal and at its termination in the inferior or superior location, respectively. A soft tissue reformatted scan was used to determine the distance between the optic canal and the termination of the nerve at the globe for the right and left eyes, respectively. This value was used to determine the linear percentage of filling of the nerve (Figures 1A,B). At completion of the studies, the animal was euthanized per the accepted recommendations of the Panel on Euthanasia of the American Veterinary Medical Association.

## Results

Pig #1 (female): Contrast (Iopamidol 41% concentration) was administered into the CSF space in the lumbar spine. Serial CT imaging identified contrast accumulation in the lumbar spine without migration. After 30 min, the pig was placed in Trendelenburg position (~15°) and the animal was re-imaged. Another 14 min later, the pig's hind region was raised up to ~30°. Contrast eventually migrated into the chiasmatic cistern at 55 min after contrast administration and 22 min after body re-positioning. After serial scanning, ending at 64 min, no contrast was observed entering the perioptic subarachnoid space, even though the contrast was situated in the chiasmatic cistern.

Pig #2 (female): Contrast was administered at a higher concentration (Iopamidol 76%) compared to the pig #1 trial. After injection of contrast, the hind region of the pig was minimally elevated to an acute angle of nearly 5–10° and the head of the pig tilted laterally, and serial CT imaging was performed. Contrast was observed to migrate rostrally through the spinal column to the prechiasmatic cistern with entry into the perioptic subarachnoid space in the dependent position at 14 min (Figure 1C). Six minutes after observed entry into the orbital optic nerve, there was complete filling of the dependent optic nerve, and partial filling of the non-dependent optic nerve. It is noted that first detection of contrast within the optic nerve revealed ~52.3% of the inferior aspect, and 22.7% of the superior aspect of the nerve had been entered. There was 13.6% entry in the non-dependent POSAS (Table 1).

**Table 1:** Experiment #2: Estimated migration of contrast front vs. time of injection of contrast agent (iopamidol 370).

**Experiment #2**	
**Time from Injection (minutes)**	**Location of contrast front**
0	Injection in lumbar spine
4	–
7	Near optic chiasm
9	Near optic chiasm
14	Dependent POSAS entry (52.3% inferior, 22.7% superior), 13.6% in non-dependent POSAS
20	Complete filling of dependent POSAS 32.6% filling of non-dependent POSAS

Pig #3 (female): Administration of contrast of iopamidol 76% via lumbar puncture was performed. The hind region of the pig was similarly elevated as before with the head rotated with the right orbit ~1 cm below the left on the horizontal axis. Serial CT imaging was performed, and contrast was observed to reach the chiasmatic cistern. Contrast was visualized to have entered into the dependent optic nerve and migrated 82.4% of the length of the nerve at 55 min post-injection. The contralateral, non-dependent nerve had 37.6% entry of the inferior aspect of the nerve and ~31.3% entry into the superior aspect of the same nerve. At 58 min, there was complete filling of the dependent perioptic subarachnoid space, and 59.2% inferior and 36.8% superior filling of the non-dependent nerve sheath (Figure 1D, Table 2).

**Table 2:** Experiment #3: The pig's head was rotated such that the right orbit was dependent and left orbit was non-dependent.

**Experiment #3**		
**Time from Injection (minutes)**	**Location of contrast front**	
	**Right (Dependent)**	**Left (Non-dependent)**
0	Injection in lumbar spine	
55	82.4%	37.6% inferior, 31.3% superior
56	97.3%	44.0% inferior, 30.8% superior
57	101.4%	53.4% inferior, 31.4% superior
58	101.3%	59.2% inferior, 36.8% superior

*Contrast migration described as an estimate of its length across the optic nerve. In the left orbit, there was differential filling of the inferior aspect of the nerve compared to the superior aspect of the same nerve. The measured linear distance between the optic canal and the right and left globes were 28.91 and 27.55 mm, respectively*.

The head was rotated to convert the non-dependent nerve into the dependent space and serial imaging ensued. At 63 min, the first scan after head tilt, 75.1% of the inferior and 61.7% of the superior aspect of the nerve were filled. Seven minutes later, the nerve had been completely filled inferiorly, although the front did not migrate much further on the superior aspect of the perioptic subarachnoid space (~61–70%; Table 3). There was, however, filling of the bulbous cul de sac of the nerve which did fill the most proximal superior aspect of the nerve (Figure 1E).

**Table 3:** Experiment #3 continued: Following scanning at the 58th min, the head was rotated so that the left orbit was made dependent.

**Head tilt**	
**Time from Injection (minutes)**	**Location of contrast front**
	**Left (Dependent)**
63	75.1% inferior, 61.7% superior
64	75.5% inferior, 64.8% superior
65	83.1% inferior, 66.5% superior
67	91.1% inferior, 69.3% superior
69	91.7% inferior, 67.7% superior
70*	104.2% inferior, 72.6% superior (Bulbous cul de sac filling)
73*	98.4% inferior, 68.5% superior (Bulbous cul de sac filling)

Scanning was repeated in the documented intervals and estimated contrast front location described in terms of the percentage of the length of the nerve. The measured linear distance between the optic canal and the right and left globes were 28.91 and 27.55 mm, respectively. *Effectively complete filling.

## Discussion

This porcine model allows for the imaging of contrast migration through the subarachnoid space as a function of time without the restriction of radiation exposure from repeated CT imaging. The model was able to provide information about contrast migration into the orbital CSF space. Preliminary results revealed features of contrast-CSF migration as well as limitations of studying CSF flow with a heavy contrast agent. In the absence of eye movement, it takes at least 6 min for contrast to migrate down the perioptic subarachnoid space to the globe, appreciated in two of the pigs. Eye movement would be expected to accelerate CSF and contrast movement. There is a dependent component to CSF migration at least as studied by this poorly diffusible contrast agent. Our results suggest that flow of contrast preferentially accumulates at 1/4th to 1/3rd down the length of the optic nerve upon its initial entry into the orbit. It is interesting to note that the contrast front from Killer's study, identifying reduced flow in the POSAS of NTG patients, was similar to that seen in this study in a pig model (10). Based on these preliminary data and Killer's prior work, we believe that the nerve sheath, as it approaches the optic canal with its known decreased compliance and the increased trabeculations within, act as a complicated extension of the canal's flow restriction.

Furthermore, we suggest four hypotheses that may explain the observed phenomenon:

- CSF is incompressible; hence, its volume flow rate due to clearance into the venous system (16) must be the same in any cross-section. If the flow passes through a system of canals and cavities with different cross-sectional areas, the mean flow velocity through a passage with a larger cross-section must be smaller than the mean flow velocity through a passage with a smaller cross-section. The slowdown of the contrast velocity may thus be explained by an increase of the effective cross-sectional area 1/4th to 1/3rd down the length of the optic nerve.
- The slowdown of the contrast velocity may also be explained by an increase of the rate of reabsorption of CSF in the region where contrast accumulates, which reduces the CSF velocity.
- The third possible explanation is that the CSF flow may be time-dependent, and the flow rate reduces by the time the contrast reached 1/4th to 1/3rd down the length of the optic nerve. The reduction of the CSF velocity may be explained by a gradual increase of hydraulic resistance to the CSF flow. For example, Rodbard (17) studied flow in collapsible tubes and demonstrated that in a completely distended tube, the increase in the outflow resistance led to a decrease in the flow rate through the tube. Another possible reason for the increased hydraulic resistance could be gradual reduction in the permeability of the optic nerve subarachnoid space. Studies have revealed that the optic nerve subarachnoid space is characterized by broad septae and small trabeculae along its length (10, 18, 19). This leads to the consideration that the flow of cerebrospinal fluid through the subarachnoid space can be modeled as a flow through a porous medium (16, 20). A decrease in the permeability of the optic nerve subarachnoid space could be causing a reduction in the CSF flow rate.
- The fourth possible explanation is based on interaction between flows of a denser contrast and a less dense CSF. For example, due to local CSF circulation the rate of contrast front propagation may not be representative of CSF velocity, and CSF may bypass or channel though the accumulating contrast layer.

The optic canal is nearly 10 mm in length, and the subarachnoid space, if patent, can be <1 mm. Additionally, the optic nerve SAS is characterized by numerous trabeculae and septae (18, 19). In essence, this thin area acts as a flow restrictor and the flow through it can be simulated using Darcy's law describing fluid flow through a porous medium (20);

$$\begin{array}{l} Q=\;-\frac{kA\Delta P}{\mu l} \end{array}$$

where *k* is the permeability, Δ*P* is the pressure difference, *A* is the cross-sectional area, μ is the dynamic viscosity of the fluid, and *l* is the length of the optic canal.

The viscosity of iopamidol at a concentration of 200 mgl/ml (41%) at 37°C is ~2.0. Compared to the 0.7 mmPa viscosity of cerebrospinal fluid at 37°C, theoretical flow is reduced nearly 65%. At a concentration that of Iopamidol 300 mgl/ml (61%), the flow is reduced more than 85%. Therefore, it is important to account for the fact that contrast-based studies, such as Killer's (10) and this present model, do not replicate physiologic CSF flow.

There are a number of limitations of this study. The first is that the porcine model is not an ideal example of human CSF relationships given that the animal is a quadruped with vastly different positional and dependent pressure relationships between compartments. Even if otherwise healthy, anesthetized animals are not representative of truly normal, healthy physiology. This is particularly important in that anesthesia limits eye movement, an important contributor to CSF flow in the orbit. Further, for the migration of contrast in a human CT myelogram, a certain amount of positioning, either by the patient or by the table, is required. In the anesthetized pig, the only motion of the animal itself is from induced breathing. Therefore, all movement is performed by external mechanisms. Another major limitation is the small numbers of pigs used for the investigation. This is due to the nature of invasive research in an animal model. However, we believe the successful procedures provided the information being sought out. The amount of contrast that was eventually administered in the CSF space was not recorded. Initial injections were needed to ensure the correct entry into the subarachnoid space, and therefore the overall contrast that was eventually injected was unable to be accurately measured. We did not feel that reporting estimates was appropriate.

The use of gadolinium (Gd) and MRI was considered. However, the need to develop this model in conjunction with the eventual need for invasive interventions, we felt that CT would offer the greatest access. Manipulation within the magnet would also be limited due to constraints from the coil. Furthermore, the susceptibility artifacts associated with this region could severely limit our ability to visualize the contrast front. We do anticipate exploring the magnetic resonance visualization of the model more fully in the future.

Hounsfield units were not used to determine true contrast fronts, although due to the partial volume effects in such a small space as the perioptic subarachnoid space, the ability of Hounsfield unit determination would be diminished. The ROIs for the optic nerve were determined in a linear fashion for overall simplicity and to account for potential eye movement. Therefore, the natural orbital redundancy and mild tortuosity of the optic nerve was not accounted for by this technique. There may also be other ways of having administered contrast into the intracranial CSF space which may have increased the yield of success for acquiring the CSF space in all pigs. One example is the work of Kaiser et al. (21), although the imaging laboratory where the procedure was performed was not amenable to more invasive means such as this.

Lastly, it should be reiterated clearly that radiographic examinations involving intrathecal contrast are not accurate representations of CSF flow, especially as described in this model. For reasons described above the relationships examined herein should be appreciated in the context of the significant deviations of normal physiology, fluid properties, and dynamics introduced by contrast, anesthesia, anatomy, and species.

In conclusion, we present the development of a novel porcine model for studying CSF flow in the neural axis and the orbit. Based on the preliminary data, we surmise that CSF flow in the porcine perioptic subarachnoid space is slow in the absence of eye movement, and likely much slower than measures of CSF in other commonly tested parts of the central nervous system, like the cerebral aqueduct (22). As speculated by others, the optic canal is likely acting as a flow restrictor, and this may be accentuated by contrast agents that have higher intrinsic viscosity compared to cerebrospinal fluid. Gravitational dependence is also important in the flow of CSF, consistent with our understanding of the influence of hydrostatic pressure in our measurement of intracranial pressure (23). Lastly, while this is speculative based on the few pigs studied, there seems to be preferential filling of the first 1/4th to 1/3rd of the optic nerve sheath before it begins to slow down in filling the remainder of the orbital CSF space. This may explain a prior group's identification of a contrast front in the same region in a patient with NTG. This model will be used for future work that will control the amount of contrast, positioning, artificial eye movement, and other physio-anatomic parameters to perform a robust investigation of physiologic CSF dynamics.

## Data Availability Statement

All datasets generated for this study are included in the manuscript/supplementary files.

## Ethics Statement

The animal study was reviewed and approved by University of North Carolina at Chapel Hill IACUC.

## Author Contributions

DF: design of study, conduction of experiment, data analysis, and author of manuscript. OK, BT, AK, and LG: data analysis and contributor to manuscript. RS: data analysis. XZ, DO, and CZ: conduction of experiment. YL: design of study, conduction of experiment, data analysis, and contributor to manuscript.

### Conflict of Interest

The authors declare that the research was conducted in the absence of any commercial or financial relationships that could be construed as a potential conflict of interest.
